# Effectiveness of Mindfulness-Based Group Therapy Compared to the Usual Opioid Dependence Treatment

**Published:** 2015-06

**Authors:** Saeed Imani, Mohammad Kazem Atef Vahid, Banafsheh Gharraee, Alireza Noroozi, Mojtaba Habibi, Sarah Bowen

**Affiliations:** 1 Department of Clinical Psychology, Shahid Beheshti University, Tehran, IR Iran; 2 Department of Clinical Psychology, Tehran Psychiatric Institute, Iran University of Medical Sciences, Tehran, IR Iran; 3 Iranian National Center for Addiction Studies (INCAS), Iranian Institute for Reduction of High-Risk Behaviors, Tehran University of Medical Sciences, Tehran, IR Iran; 4 School of Advanced Technologies in Medicine, Tehran University of Medical Sciences, Tehran, IR Iran; 5 Head of Substance Abuse Prevention and Treatment (SAPTO), Iran MoH; 6 Family Research Institute, Shahid Beheshti University, Tehran, IR Iran; 7 Addictive Behaviors Research Center, Department of Psychology, University of Washington, Seattle, USA.

**Keywords:** *Mindfulness*, *Group Therapy*, *Opioid*

## Abstract

**Objective: **This study investigated the effectiveness of mindfulness-based group therapy (MBGT) compared to the usual opioid dependence treatment (TAU).Thirty outpatients meeting the DSM-IV-TR criteria for opioid dependence from Iranian National Center for Addiction Studies (INCAS) were randomly assigned into experimental (Mindfulness-Based Group Therapy) and control groups (the Usual Treatment).The experimental group undertook eight weeks of intervention, but the control group received the usual treatment according to the INCAS program.

**Methods:** The Five Factor Mindfulness Questionnaire (FFMQ) and the Addiction Sevier Index (ASI) were administered at pre-treatment and post-treatment assessment periods. Thirteen patients from the experimental group and 15 from the control group completed post-test assessments.

**Results:** The results of MANCOVA revealed an increase in mean scores in observing, describing, acting with awareness, non-judging, non-reacting, and decrease in mean scores of alcohol and opium in MBGT patient group.

**Conclusion:** The effectiveness of MBGT, compared to the usual treatment, was discussed in this paper as a selective protocol in the health care setting for substance use disorders.

According to a national epidemiologic study conducted by Ministry of Health (33), there are more than 1.2 million opioid dependents living across the country who use drugs usually smuggled into the country from Afghanistan. Currently, opioid maintenance treatment programs and short-term medically assisted withdrawal are delivered through a network of more than 2500 certified addiction treatment professionals at the national level. Although delivering psychosocial services in combination with medical management was emphasized in national methadone protocol (26), there is a scarcity of evidence in evidence-based psychosocial intervention for improving medical treatment outcomes. 

Mindfulness is a technique comprised of meditation and a specific mental tendency toward experience that encourages an awareness of the present-moment experience. This awareness is non-evaluative and minimizes over-involvement with thoughts and emotions (28). In contrast to opioid maintenance treatment which has proved less effective in reducing the stress-induced craving (21), new approaches of psychotherapy that focus on mindfulness and acceptance might hypothetically enhance anti-craving properties of opioid maintenance treatment through reduction of stress-induced craving. Although the content of cognitive distortions constitute the main focus of cognitive behavioral therapies, mindfulness focuses primarily on assisting clients in examining their relationship with their thoughts. Mindfulness encourages patients to learn to monitor their own mental processes free of judgment and without being engaged in the actual content of their thoughts. The capability to respond to aversive cognitions, sensations, and emotions with an attitude of nonjudgmental, acceptance, and present-moment awareness forms the primary skill in mindfulness (29). Through mindfulness practices, the content of the thought becomes less important than the extent to which the individual is aware of it and how they react to the occurrence of the thought (29).

Mindfulness-based relapse prevention (MBRP) (5) is a program integrating mindfulness meditation practices with traditional relapse prevention (RP) whose effectiveness has been confirmed in different populations of drug users (4). Similar mindfulness-based programs for substance abuse have proved to increase compliance, cognitive control, and emotion regulation and reduce stress among alcohol dependents (12, 14, 17) and also affect various other related factors (8). The effectiveness of mindfulness in different populations of drug users suggests that more trials, in which the type of Mindfulness Based Group Therapy is clearly defined and described, are required to determine the efficacy of Mindfulness-Based Group Therapy for opioid dependence treatment. 

The present study aimed to assess the effectiveness of Mindfulness-Based Group Therapy compared to the usual treatment for opioid dependence among different populations of opioid users.

## Material and Methods

*Participants*

 The opioid dependent clients who were receiving usual treatment in Iranian National Center for Addiction Studies (INCAS) were randomly assigned into intervention and control groups. INCAS Clinic, located in the southwest of Tehran, the capital of Iran, provides assisted withdrawal and maintenance treatment with opioid agonist medications including methadone and buprenorphine to its opioid dependent clients (96.6% male. 

Eligibility criteria were as follows: a) diagnosis of opioid dependence according to DSM-IV-TR criteria, b) 18 to 40 years of age, c) at least 8 years of completed education, d) completion of two weeks of medical treatment with opioid agonist medication and e) informed written consent for participation in the study. The exclusion criteria were psychosis, dementia, imminent suicide risk, organic brain disorders, or diagnosis of other drug dependence (except nicotine).

*Interventions*

**Figure 1:**
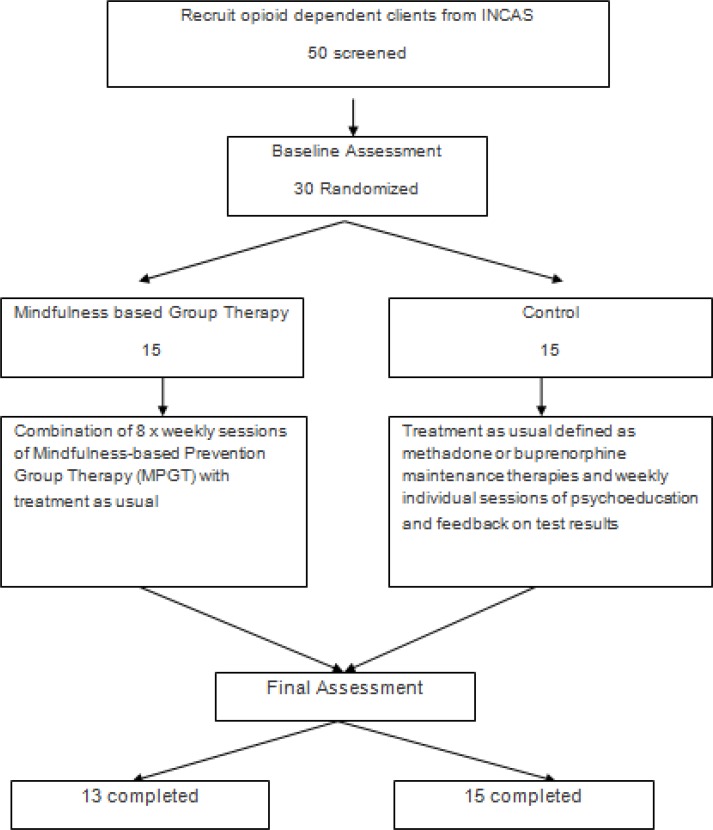
Flow chart of clinical trial of MPGT and group control treatment of methamphetamine dependence

Intervention was a combination of eight sessions of mindfulness-based group therapy developed by Bowen et al. (5) and the usual treatment service for opioid dependents in INCAS academic clinic which consisted of medical management and weekly individual counseling sessions to provide psychoeducation and feedback on the results of weekly urine tests in autumn 2013 (Figure 1).

The study was first designed in a manner that would allow comparison with other studies conducted on mindfulness-based group therapy for addiction treatment. An English manual on the structured intervention (5) was first translated into Farsi by one of the authors (SI) of this paper and then edited by a Ph.D. of clinical psychology (BG) as an addiction expert. The therapy sessions were in a closed-group format and were conducted on a weekly basis. Each session lasted for 120 minutes. The intervention was structured and had a predefined title and content for each session, and thus sessions were kept in sequence. Table 1 outlines an overview of the topics and themes that were covered in each session. Although each session had a central theme, the sessions were intended to build upon previous weeks’ practices. Details of the sessions are presented in Table 1. 

All study participants received medical treatment with opioid agonist medications in outpatient INCAS clinic according to the national protocols (26). Methadone or buprenorphine was prescribed through regular visits by a certified physician. The visits were more frequent during the first weeks of treatment and then decreased to at least weekly visits. Clients who received maintenance treatment had to come to the clinic for daily-supervised dosing during the first two months of the treatment. The take-home doses were allowed one day per week from start of the third month and could be increased from one day per month to at most 6 days a week contingent to appropriate compliance and negative weekly urine tests for morphine and methamphetamine. 

*Objectives*

This study was a pilot, randomized, parallel group, one-to-one controlled trial investigating feasibility, acceptability and effectiveness of mindfulness-based group therapy in improving treatment outcomes in opioid dependent clients.

*Instruments*

*Addiction Severity Index (ASI)* *:*

Addiction Severity Index (5th Edition) (25) was employed to assess the clinical condition of the patients. ASI is a semi-structured interview that is conducted by trained researchers. This questionnaire collects data on patients’ problems in all areas and within the last 30 days, last year and during their lifetime. ASI yields a total combined score (1-0) which grades each patient’s condition in each of the areas.

This questionnaire contains 116 items and 8 sub-scales on patients’ medical condition, vocational condition, drug and alcohol abuse, income condition, family condition, and mental condition. ASI was administered twice with a one-week interval on 20 male opioid-dependent patients who were receiving treatment. Stabilities of 0.91, 0.84, 0.83, 0.76, 0.68 and 0.73 were obtained through test-retest for the above subscales, respectively (1).

The Five Factor Mindfulness Questionnaire (FFMQ):

The ﬁve factors of mindfulness are measured by a 39-item scale called Five Factor Mindfulness Questionnaire (2). These five factors are as follows: a) Observing (attending to or noticing internal and external stimuli, such as feelings, cognitions, sights, sounds, and smells); b) Describing (noticing or mental word-tagging of these stimuli); c) Acting with awareness and consciousness (attending to one's current actions in contrast to behaving automatically or unconsciously); d) Non-judging the inner experience (abstaining from assessment of one's feelings, cognitions, and emotions); (e.) Non-reactivity to the inner experience (allowing thoughts and feelings to come and go, without being engaged in them). Responses to the items fall on a 5-point Likert-type scale (1= never or very rarely true, 5 = very often or always true). The ﬁve subscales exhibit an adequate to good internal consistency (2). To use this scale for an Iranian population, similar steps were taken. The test-retest was administered on 30 participants with a 2-week interval and showed a consistency of 0.74 between the two administrations and proved significant at the level of 0.001. The Cronbach’s alpha coefficient of the test stood at 0.89 as well. 

*Sample Size Calculation*

This was a pilot, proof of concept study to test the feasibility and efficacy of MBGT among opioid dependent clients. Thirty cases were selected randomly for participation in the study, and were then assigned equally to each intervention group. All cases filled out written consent form to participate in the study. The intervention and condition groups shared basic similarities in socio-demographic variables. In order to have consistency in seasonality across assessment points, the assessment conducted in Fall 2013 served as the baseline or pre-test assessment point for the main substantive intervention outcome analyses, and the Winter 2013 assessment served as the outcome stage point for the analyses report. Hence, the design of the current research was of an experimental nature. 

*Statistical Analysis*

Treatment analysis was intended to compare the outcomes obtained for the two groups. To determine the legitimacy of the numerical codes, data cleaning was performed to ensure data accuracy. 

Both graphical and descriptive statistics were conducted to check for out-of-range values for categorical variables, and mean and standard deviations were in a plausible range for continuous composite variables (30). The patterns of missing data, the quantity of missing data and the reason they were missing were also checked. The result of SPSS MVA (missing values analysis) using t test (α = 0.05) 

for the outcome of composite variables was checked to ensure that data loss patterns in outcome variables were not related to participants’ demographic variables such as gender, area of residency, and group (intervention vs. condition). Distribution of scores on continuous variables were also checked for univariate (p<0.001) and multivariate normality using LISREL, version 8.72 (22). To match the intervention and control groups on demographic variables such as age, gender, education, marital status and employment status, t test and Chi-square test were conducted. Repeated measure analyses of variances were utilized to test the effectiveness of Mindfulness-Based Group Therapy in the participants.

## Results

The experimental and control groups were matched in terms of age, education, marital status and job status (Table 2).

**Figure 2:**
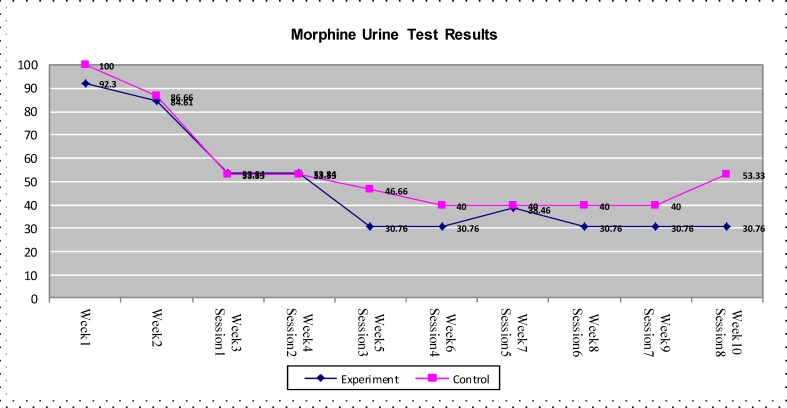
Results of morphine urine test in both experimental and control groups during ten weeks

Figure 2 demonstrates that according to the addiction test, a decrease of opioid consumption was witnessed in both groups, but the experimental group showed a more significant decrease than the control group (P<0.01). 

**Table 1:** Mindfulness-Based Relapse Prevention Group Therapy Sessions

**Session No. **	**Central Theme **	**Session Details**
Session One:	Automatic Pilot And Relapse	Session 1 was specifically in relation to addiction, its consequences and emotional disturbances. This exploration began with an exercise called “body scan.”
Session Two:	Awareness Of Triggers And Craving	Session 2 was on learning to experience triggers, thoughts and cravings without reacting “automatically.”
Session Three:	Mindfulness In Daily Life	Session 3 dealt with learning the "SOBER space" practice as a way to expand the quality of mindfulness, from formal sitting or lying down practice to daily situations.
Session Four:	Mindfulness In High Risk Situation	Session 4 was on mindfulness practice in high-risk situations, with a focus on being present in circumstances or with people that have previously been associated with or led to substance use. It also dealt with learning to experience pressures or urges without automatically reaching for a substance.
Session Five:	Acceptance And Skillful Action	Session 5 was about balancing acceptance and skillful action, exploring the seeming paradox of accepting unwanted thoughts, feelings, and sensations, and learning the role of acceptance in the change process.
Session Six:	Seeing Thoughts As Thoughts	Session 6 explored the nature of mindfulness and its relation to thinking, with a focus on experiencing thoughts as merely thoughts.
Session Seven:	Self-Care and Lifestyle Balance	Session 7 was on balancing self-care and lifestyle, with a focus on personal warning signs for relapse and how to best respond when these warning signs arise.
Session Eight:	Social Support And Continuing Practice	Session 8 dealt with social support, continuing practice and the importance of building a support system.

**Table 2:** Comparison of Experimental and Control Groups Based on Demographic Variables

	Experimental N=15		Control N=15			p-values
Age	Mean	S.D.	Mean	S.D.		
	38.69	4.38	36.13	5.09	t(26)= 1.41	p>.05
Marital Status	f		f			
Married	6	(46%)	8	(53%)		
Single	4	(31%)	5	(33%)		
Divorced	3	(23%)	2	(14%)	χ2 (2)= 0.46	p>.05
Job Status						
Unemployed	11	(15%)	9	(6%)		
Employed	2	(85%)	6	(4%)	χ2 (1)=2.07	p>.05
Educational Level						
Higher Education	1	(8%)	1	(7%)		
Diploma	12	(92%)	14	(93%)	χ2 (1)= 0.01	p>.05

**Table 3:** Results of MANOVA for Experimental and Control Groups in Mindfulness Subscales in pretest assessment

**Subscales**	**Variable**	**Degree of** **Freedom**	**F Value**	**Significance** ** Level**	**Effect Size**
Observing	Five Factor Mindfulness Questionnaire	1-21	0.91	0.34	0.03
Describing		1-21	0.01	0.90	0.00
Acting with awareness		1-21	3.55	0.07	0.12
Non-judging		1-21	0.18	0.67	0.00
Non-reactivity		1-21	1.33	0.25	0.05

**Table 4:** Results of MANCOA for Experimental and Control Groups in Mindfulness Subscales with adjustment of pretest scores on posttest scores

**Subscales**	**Variable**	**Degree of** **Freedom**	**F Value**	**Significance** ** Level**	**Effect Size**
Observing	Five Factor Mindfulness Questionnaire	1-21	70.24	0.001	0.77
Describing		1-21	114.59	0.001	0.84
Acting with awareness		1-21	264.87	0.001	0.92
Non-judging		1-21	5.90	0.024	0.22
Non-reactivity		1-21	49.52	0.001	0.70

**Table 5:** Results of MANOA for Experimental and Control Groups in Addiction Sevier Index in pretest

**Subscales**	**Variable**	**Degree of** **Freedom**	**F Value**	**Significance** ** Level**	**Effect Size**
Alcohol	ASI	1-24	0.91	0.76	0.07
Opiom		1-24	0.60	0.82	0.06

**Table 6:** Results of MANCOVA for Experimental and Control Groups in Addiction Sevier Index with adjustment of pretest scores on posttest scores

**Subscales**	**Variable**	**Degree of** **Freedom**	**F Value**	**Significance** ** Level**	**Effect Size**
Alcohol	ASI	1-24	15.05	0.001	0.44
Opiom		1-24	23.06	0.001	0.54

**Figure 3:**
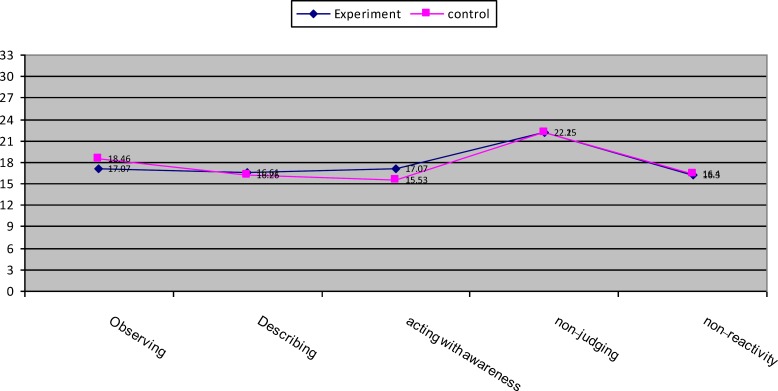
Mean score profile for experimental and control groups in mindfulness subscales in pretest assessment

**Figure 4:**
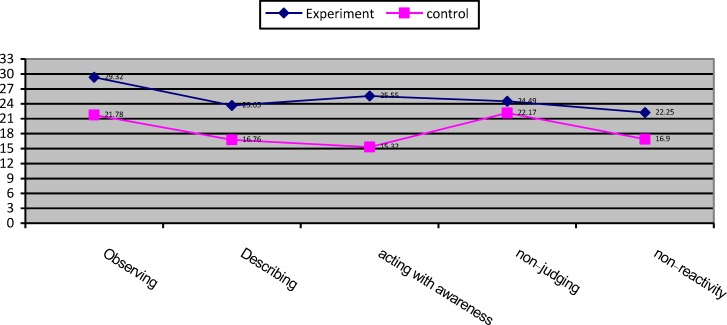
Mean score profile for experimental and control groups in mindfulness subscales pretest scores on posttest scores.

**Figure 5:**
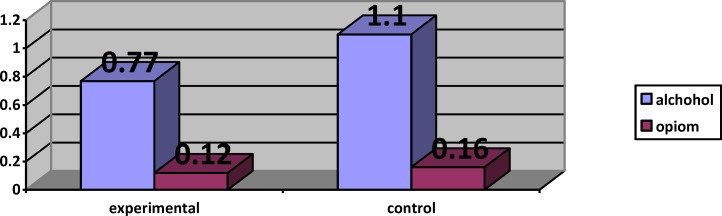
The comparison of ASI subscales (alcohol and opium) in both experimental and control

**Figure 6:**
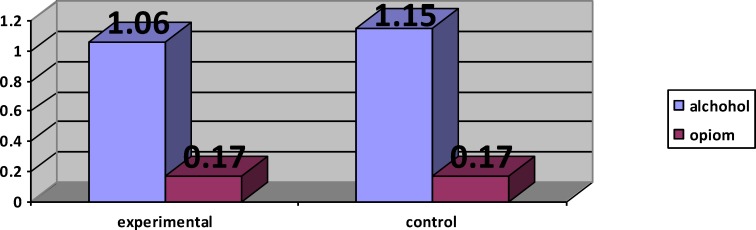
The comparison of ASI sub scales (alcohol and opium) in both experimental and control

The analysis of MANCOVA revealed that the experimental group had a higher mean score in observing (F(1,21) = 70.24, P<0.001, η2 = 0.77), describing (F(1,21) = 111.59, P<0.001, η2 = 0.84), acting with awareness (F(1,21) = 264.87, P<0.001, η2 = 0.92), non-judging (F(1,21) = 5.90, P<0.05, η2 = 0.22), non-reactivity (F(1,21) = 49.52, P<0.001, η2 = 0.70) compared with control group (Tables 3 & 4, Figures 3 & 4). The covariance analysis results of ASI confirms the assumed homogeneity of variance – covariance matrix (F(2244,28.99) =1.04, p>0.05). Evaluation of the intervention effect on each of the subscales along with controlling the pre-test effect as the covariance factors on post-test indicated that ASI in the experimental group (M=0.77) in the alcohol subscale of control group was (M = 1.10) (F(21,1)-0.66, P<0.001), and the ASI of the intervention group (M = 0.12) in the drug subscale of the control group was (M = 0.16) (F21, 1)-0.01, P<0.001) (Tables 5 & 6, Figures 5 & 6).

## Discussion

Results of this study provided preliminary evidence supporting the feasibility and efficacy of adding-on MBRP to the usual treatment compared to the treatment as usual alone among opioid dependent clients. Outcomes revealed a more significant improvement in drug section of ASI and weekly morphine urine tests in MBRP group compared to those receiving the usual treatment.

Differences were evident in all sub- scales of mindfulness including observing, describing, being nonjudgmental of inner experience and no reactivity to inner experience (9, 18, 20, 31, 32).

Compared to the usual treatment, Mindfulness proves to have a positive effect. Research findings suggest the effectiveness of mindfulness techniques in treatment of opioid dependents. Results obtained by other research works confirm the results of the current study. The current findings along with findings of other researchers indicate that usual therapies, which are mainly performed by medicine and methadone, are not able to treat patients’ dependency on opioids, and psychiatric interventions such as mindfulness can play an important role in the treatment of dependence on opioids and prevention of relapse (11, 20(.

 The findings of the current study on the improving the effect of mindfulness correspond to those obtained by Breslin, Zack and McMain (2); Dabaghi, Asgharnejad, Atef Vahid and Bolhari (10); Leigh, Bowen and Marlatt (24); Bowen, Witkiewitz, Dillworth and Marlatt (3); Edward (11); Garland, Gaylord, Boettiger, Matthew and Howard (16). Reviewed studies indicated mindfulness meditation as a useful additional strategy for reducing relapse (6). Previous studies have shown that attempts to suppress thoughts about substance abuse can result in an increase in actual abuse. Mindfulness interventions emphasize acceptance, rather than suppression of unwanted thoughts. In case of success, this decreases the meaningful rate of thought (3). 

Mindfulness training might disrupt the chain risk of stress-precipitated substance relapse. Mindfulness training reduced stress and thought suppression significantly, and increased recovery from substance cues (16). The long-term effectiveness of mindfulness meditation as a treatment is proved for relapse prevention (34).

Moreover, participation in mindfulness-oriented interventions is associated with significant increases in mindfulness that in turn mediates the effect of mindfulness training on reduction of psychological symptoms (7). Thus, mindfulness intervention training (4, 16) may produce salutary effects on addictive processes through their promotion of mindfulness.

The obtained results indicate the efficacy of mindfulness based therapy for decreasing the ASI in alcohol and drug consumption. The obtained results confirm those achieved by Dabaghi, Asgharnejad, Atef Vahid and Bolhari (10); Edward (11); Garland, Gaylord, Boettiger, Matthew and Howard (16); Morone, Lynch, Losasso and Liebe (27). 

To clarify the results, it can be stated that mindfulness-based intervention can decrease addictive behaviors towards alcohol and drugs (15) and disrupt stress-relapse cycle and as a result decreases drug consumption, stress and thought suppression significantly and improves the recovery rate (16). It also improves the cognitive control over temptation and decreases stress and anxiety attached to alcohol and drug consumption (13, 14, 23).

There were some limitations regarding the present study that merit discussion. Sessions were not recorded to be randomly reviewed for fidelity purposes. The small sample size of the study and using only one consultant were among other limitations of the study which need to be taken into consideration in future studies. A larger sample and dependency on other substances from other groups can also be considered for future studies to assess whether the findings can be generalized to a larger population.

## Limitations

 The sample size of the study was small and further studies with larger sample size to explore the effects of clients characteristics on the intervention outcome is suggested. 

## Conclusion

Due to low mindfulness in addicts, especially after repetitive relapses, identification of risk factors and requirements among addicts can increase their mindfulness by performing related interventions. Mindfulness was considered as one of the major factors in the current study. Individual, familial, occupational, financial and social side effects of addiction have motivated individuals, families and authorities to seek experts’ advice to prevent symptoms and relapse of this condition. 

Providing training and promoting mindfulness as an effective factor for the treatment and reduction of detrimental impacts of addiction can be a major step toward treatment of dependency on drugs and its individual and social impacts. Through providing adequate trainings to patients, the addiction intensifying factors can be harnessed, preventive measures can be strengthened, and the forthcoming detrimental outcomes can be prevented. Thus, the results of this research may be used by mental health experts, families etc.
